# Leveling the cost and carbon footprint of circular polymers that are chemically recycled to monomer

**DOI:** 10.1126/sciadv.abf0187

**Published:** 2021-04-09

**Authors:** Nemi Vora, Peter R. Christensen, Jérémy Demarteau, Nawa Raj Baral, Jay D. Keasling, Brett A. Helms, Corinne D. Scown

**Affiliations:** 1 Biological Systems and Engineering Division, Lawrence Berkeley National Laboratory, Berkeley, CA 94720, USA.; 2 Advanced Systems Analysis Group, International Institute for Applied Systems Analysis, Schlossplatz 1, A-2361 Laxenburg, Austria.; 3 The Molecular Foundry, Lawrence Berkeley National Laboratory, Berkeley, CA 94720, USA.; 4 Life-Cycle, Economics, and Agronomy Division, Joint BioEnergy Institute, 5885 Hollis Street, Emeryville, CA 94608, USA.; 5 Department of Chemical and Biomolecular Engineering, University of California, Berkeley, Berkeley, CA 94720, USA.; 6 The Novo Nordisk Foundation Center for Biosustainability, Technical University of Denmark, Lyngby, Denmark.; 7 Center for Synthetic Biochemistry, Institute for Synthetic Biology, Shenzhen Institutes for Advanced Technologies, Shenzhen, China.; 8 Biofuels and Bioproducts Division, Joint BioEnergy Institute, 5885 Hollis Street, Emeryville, CA 94608, USA.; 9 Materials Sciences Division, Lawrence Berkeley National Laboratory, Berkeley, CA 94720, USA.; 10 Energy Analysis and Environmental Impacts Division, Lawrence Berkeley National Laboratory, Berkeley, CA 94720, USA.; 11 Energy & Biosciences Institute, University of California, Berkeley, Berkeley, CA 94720, USA.

## Abstract

Mechanical recycling of polymers downgrades them such that they are unusable after a few cycles. Alternatively, chemical recycling to monomer offers a means to recover the embodied chemical feedstocks for remanufacturing. However, only a limited number of commodity polymers may be chemically recycled, and the processes remain resource intensive. We use systems analysis to quantify the costs and life-cycle carbon footprints of virgin and chemically recycled polydiketoenamines (PDKs), next-generation polymers that depolymerize under ambient conditions in strong acid. The cost of producing virgin PDK resin using unoptimized processes is ~30-fold higher than recycling them, and the cost of recycled PDK resin ($1.5 kg^−1^) is on par with PET and HDPE, and below that of polyurethanes. Virgin resin production is carbon intensive (86 kg CO_2_e kg^−1^), while chemical recycling emits only 2 kg CO_2_e kg^−1^. This cost and emissions disparity provides a strong incentive to recover and recycle future polymer waste.

## Teaser

While not yet competitive with commodity polymers, PDKs are recycled to monomer at lower cost and emissions than virgin resins.

## INTRODUCTION

Life cycles for consumer products made from polymers are overwhelmingly linear and follow a make-take-discard model (*1*). China, one of the largest global importers of waste, has implemented increasingly restrictive policies banning a majority of polymer and soiled waste imports to protect their waste disposal facilities and environment from large quantities of low-value, contaminated waste. Brooks *et al*. (*2*) project that the import bans will displace 111 metric tons (MT) of polymer waste by the next decade if current trends continue. Stringent requirements for exported waste have already caused cascading global impacts as sufficient infrastructure does not currently exist elsewhere to recycle this additional volume. Although the European Union has instituted recycled content requirements for flexible packaging (*3*) and many private companies voluntarily set goals for using recycled polymer resin, recycled polymer resins are difficult to come by in high quality and high volume, and most are unable to compete purely on price with their virgin petroleum–derived counterparts given low petroleum prices (*4*). As a result, 92% of the collected waste in the United States and 69% across Europe are diverted to landfills or waste incineration plants (*5*, *6*).

Nearly all polymer recycling conducted today is mechanical and dominated by polyethylene terephthalate (PET) and high-density polyethylene (HDPE) owing to the high molecular weight of the polymer chains used in the progenitor resins and the volume of polymer that can be collected and sorted at recycling facilities (*5*). During mechanical recycling, however, polymer chains undergo extensive chain scission. Accordingly, only 10% of polymer waste is mechanically recycled more than once, and, in most cases, mechanically recycled polymer resin requires mixing with virgin resin to maintain quality standards (e.g., melt rheology) for conversion. Quality standards are also difficult to maintain when additives [e.g., plasticizers, flame retardants, pigments, light, and heat stabilizers (*7*)] are compounded into the resin, as they are not generally removed during the production of flakes or filtered out during melt extrusion of resin pellets. Together, it is evident that the most widely practiced process for recycling polymer waste may reduce the demand for virgin resin but will not eliminate it and only delay the waste ending up in the landfill by a few years **(***8*, *9*).

Chemically deconstructing polymer waste back to monomer is a promising alternative that has been applied to PET, HDPE, polystyrene (PS), and nylon-6 (*10*). Chemical recycling to monomer encompasses a range of pyrolytic, catalytic, or enzymatic depolymerization processes as well as the chemical separations needed to refine the chemical feedstocks for reuse. Both are energy and carbon intensive and generate waste but have the advantage of recirculating most of the refined carbon for substantially longer periods in circular manufacturing systems (*11*). Aiming to lower the intensity of both chemical depolymerization and refinement, a new generation of more chemically recyclable polymers has emerged. They are known by several names, including dynamic covalent polymer networks, covalent adaptable networks, and vitrimers for the specific case of glass formers (*12*, *13*). These resins are unique in that they feature dynamic covalent bonds that allow polymer resins to be thermally processed much like thermoplastics with certain performance advantages typical of thermosets due to their networked architecture. The dynamic covalent character of the bonds comprising the networks allows most to be solvolyzed to small molecules or oligomers; however, in nearly all cases, the recovered monomers cannot be directly repolymerized to fully networked resins with similar properties. The exception to this is found in dynamic covalent polymer networks based on the chemistry of polydiketoenamines (PDKs) (*13*). PDKs self-condense from polytopic triketone and amine monomers (*14*) and are chemically depolymerized in strong acid. Each monomer can be recovered in quantitative yield in virgin quality after a filtration of the triketone monomer and a neutralization of the amine monomer. This process occurs at room temperature, substantially reducing the high energy use commonly associated with chemical recycling of commodity polymers, such as HDPE, PS, or nylon-6. In addition, their selective recovery from mixed-polymer waste is possible (*13*). Recovered monomers can be remanufactured into the same PDK resin in a fully closed loop or upcycled out of loop with other monomers to access new features without affecting their future prospects for recycling.

The properties of PDKs can be tailored through formulation to render them suitable for use in a wide range of applications, including flexible packaging for food and beverage, heat- and fire-resistant composites for consumer electronics, vehicle and aircraft parts, wind turbine blades, and energy-efficient buildings (*15*). In each of these use cases, PDKs would be adopted with the goal of diverting plastics from landfills by enabling the recycling of these products to virgin-quality monomers while not markedly increasing system-wide costs and emissions. Because PDKs and related materials are still nascent in their development and have not yet been commercialized, little is known about the costs and environmental implications of producing and recycling them, as well as the scaling challenges associated with introducing a new, more recyclable polymer resin into the market. A means to integrate process engineering, life cycle assessment (LCA), and material flow analysis is therefore needed to shed light on economic and environmental pinch points (*12*).

Here, we combine a rigorous quantitative techno-economic analysis and life cycle greenhouse gas (GHG) inventory for producing and chemically recycling PDK resins to monomer across a range of waste recovery scenarios to address the aforementioned gaps in understanding. We combine experimental data and best practices with process engineering and systems-level analysis to envision a hypothetical commercial-scale supply chain. Our life cycle framework tracks material and energy use in the entire supply chain, from raw material extraction to production at the facility and subsequent chemical recycling. We present the results in two relevant metrics: minimum selling price (MSP) and life cycle GHG emissions per unit mass of primary and recycled PDK resins. These two metrics are useful starting points, but certainly not the only relevant indicators. The goal of chemically recyclable plastics is to maximize the diversion of plastics from landfills and minimize the demand for nonrenewable materials—all at acceptable costs and emissions. However, leakage of material to the environment and the resulting fate of the plastics in different ecosystems are dependent on product use, shape, and size, and this warrants further study through streamlined experiments **(***16*) to prevent harmful effects of plastics and microplastics on the environment **(***17*, *18*).

Apart from quantifying selling price and GHG emissions, we identify pinch points in the supply chain of resin produced through recycling in comparison to resin produced from raw materials. We also assess the impact of product use lifetimes and waste resin recovery rates on the system-wide average resin MSP and GHG footprint in facilities producing both primary and circular PDK resins. Through this analysis, we determine the conditions required for PDK resins to match or outperform commodity polymers on the basis of life cycle GHG emissions and cost. While previous studies have conducted LCAs for chemical upcycling of polymer waste to out-of-loop chemical feedstocks (*19*, *20*), to our knowledge, this paper provides the first systems-level analysis of the introduction of novel circular polymer resins into the market. Furthermore, we use polymer waste recovery rates as key targets to successfully level the cost and GHG emissions, which, in turn, has implications for future sustainable manufacturing practices and supply chains.

## RESULTS

**Fig. 1:**
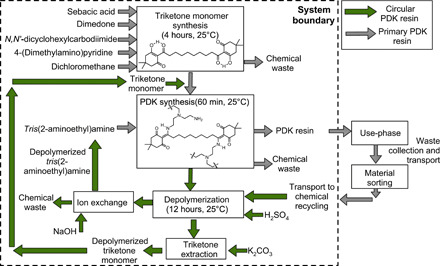
System boundary for life cycle assessment and techno-economics of primary and circular polydiketoenamine (PDK) resin production.

The build-out of a PDK resin production and recycling system requires careful coordination. Primary PDK resin production must be initially scaled up to introduce the material into products, and the timing and rate at which recovered material becomes available for depolymerization will depend largely on whether it is used for single use or durable goods. We modeled three different theoretical facilities producing PDK resins at 20,000 MT annually. The first plant produces only virgin resin using externally sourced raw materials (referred to as primary PDK resin). The second plant is a recycling-only facility that operates at a steady state and accepts postconsumer PDK waste as raw material, depolymerizes it to its constituent monomers, performs chemical separations to refine the monomers, and repolymerizes the monomers back to recycled virgin-quality PDK resin (referred to as circular PDK resin). The term “circular PDK resin” refers to the monomer’s ability to be directly repolymerized to PDK resin, which is indistinguishable from virgin material. In this second facility, any material loss during processing is made up for by sourcing additional PDK waste to maintain 20,000 MT annual output. The third facility produces a mixed PDK resin product composed of both circular PDK resin and makeup primary PDK resin to produce a total of 20,000 MT annually. In this third facility, losses in the process and incomplete recovery of postconsumer PDK are made up for with increased production in virgin PDK resin. A schematic of all the stages and inputs considered in the framework including major material requirements are presented in Fig. 1. Although we model these processes at the individual facility level, total production in the United States would need to be on the order of 10 to 100 times greater to justify recovery of PDK waste from mixed municipal streams. At smaller volumes, product take-back systems would likely be the more viable option.

### Minimum selling price of PDK resins

The MSP is defined as the PDK resin selling price required to reach a net present value of zero at the production facility after accounting for all capital and operating expenditures, as well as a 10% internal rate of return. It serves as a guidepost to understand cost-competitiveness of primary and circular PDK resins relative to conventional polymers while also identifying potential process and chemistry bottlenecks. We used chemical process simulation to estimate material and energy requirements of manufacturing PDK resins at commercial scale and used the results to estimate associated costs and revenues based on engineering project estimates and economic assumptions. We estimated that the MSP of primary PDK resins is $45/kg, and the circular PDK resins produced through recycling of PDK waste amount to $1.5/kg. These results indicate a large gap between the low cost of recycling and the comparatively high cost of primary resin production. Although this gap can provide a strong economic incentive to recover as much material as possible (for example, through product take-back systems), it also raises the question as to whether the upfront costs of producing primary PDK resins with the current chemistry identified in the discovery phase of their development will be prohibitive for market entry; we further reason that through advances in process chemistry and catalysis, this disparity can be substantially alleviated to reasonable levels for market entry.

**Fig. 2:**
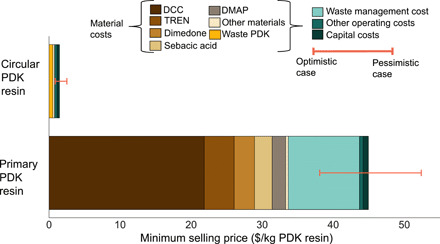
Minimum selling price contributions from primary PDK resin and circular PDK resin. DCC, *N*,*N′*-dicyclohexylcarbodiimide; DMAP, 4-(dimethylamino)pyridine; TREN, *tris*(2-aminoethyl)amine. The error bars represent a pessimistic and an optimistic case with horizontal stacked bars representing results for the baseline case.

To understand the origins of this disparity, we show in Fig. 2 the contributions from each cost component. For primary PDK resins, material costs alone contribute 75% of total MSP. The material costs are dominated by a few specialty chemicals required for triketone synthesis and polymer synthesis. This is not uncommon for chemistry demonstrated at bench scale because reactants are selected with the goal of achieving a proof of concept with minimal concern for industrial-scale process economics. In this case, the cost and use of *N*,*N′*-dicyclohexylcarbodiimide (DCC), a reactant in the synthesis of triketone monomers, comprise 49% of the total MSP. Other main cost contributions are tied to the use of *tris*(2-aminoethyl)amine (TREN) (a reactant in polymer synthesis), dimedone and sebacic acid (reactants in triketone synthesis), and 4-(dimethylamino)pyridine (DMAP) (a catalyst required for triketone synthesis)—each contributes 9, 6, 6, and 5%, respectively, to the MSP. The cost of waste management contributes 22% to the MSP because of the need to dispose of hazardous waste. The average cost of disposing hazardous waste at treatment, storage, and disposal facilities is 70 times the cost of nonhazardous solid waste disposal in the United States, where discarded solvents from chemical manufacturing are a common source of hazardous wastewater (*21*). Some wastes can be recycled, while others are treated and disposed in either landfills or incinerators. The use of DCC forms *N*,*N′*-dicyclohexylurea, a known irritant (*22*), as a by-product in triketone synthesis, requiring appropriate disposal as hazardous waste. In addition, triketone monomer synthesis requires the use of dichloromethane, which is categorized as a hazardous substance by the U.S. Environmental Protection Agency. While the plant is modeled to recycle solvents, residual chemical waste requires appropriate disposal as well (*23*). The residual waste from using DMAP is also treated as hazardous waste due to its toxicity (*24*). Operating costs other than material and waste management costs include cost of labor and cost of operating facility. The capital cost contributions to MSP are comparatively small (~2%) and include cost of purchasing and installing equipment, construction, warehouse, and engineering costs.

The production of circular PDK resins (using only recovered triketone and amine monomers) is far less costly and material intensive than primary resin production. Recovered PDK waste is depolymerized in 5.0 M sulfuric acid (reaction time of 12 hours) into triketone monomers and TREN. TREN recovery requires addition of NaOH for the ion exchange process. The subsequent polymerization is solvent free and proceeds at room temperature. As none of the input materials (K_2_CO_3_, NaOH, and H_2_SO_4_) and by-product (Na_2_SO_4_) are considered hazardous waste, we only assigned solid waste, wastewater treatment cost, and emissions to the effluent. Recovering both TREN and the triketone from PDK waste eliminates the use of DCC, DMAP, and dichloromethane that are required for triketone synthesis and other upstream emissions resulting from TREN synthesis. As a result, the cost of producing circular PDK resins is less dominated by material inputs; the material costs comprise 52% of the MSP, and capital costs are responsible for 23%. The material costs are dominated by the estimated cost of acquiring a clean waste stream; this cost alone contributes 36% to the MSP. Here, we assumed a price of $0.42 per kilogram of collected and sorted PDK waste based on recycled premium PET prices (*25*). We recognize that prices can vary by geography and other factors. Some polymer waste is sold at negative prices (i.e., receiving a tipping fee to accept recyclables) due to oil price volatility and uncertain demand.

To understand the sensitivity of the results to key uncertain parameters, we present two additional cases: an optimistic and pessimistic case. The optimistic case represents a combination of best available material prices and high reaction yields, while the pessimistic case represents a combination of higher prices and baseline reaction yields. The baseline yields were based on experimental data, while a range (low, medium, and high) of prices were used based on bulk quotes provided by chemical suppliers from online retailers. Whenever only point estimates were available, we varied the parameters using ±20% variation. The sensitivity analysis was conducted for only the relevant contributing factors identified in the baseline case including reaction yields, DCC, DMAP, TREN, dimedone, sebacic acid, and hazardous waste disposal prices. For circular PDK resins, we varied the cost of waste PDK and reaction yields. The reaction yields for monomer synthesis ranged from 90 to 95%, polymer synthesis ranged from 95 to 100%, and depolymerization ranged from 90 to 100% based on known experimental data (*13*). The highest-cost chemicals were TREN and DCC. Only one estimate of $20/kg was available for the price for TREN, and therefore, the range was varied based on ±20% variation. The price for DCC ranged from $15 to $20/kg and was developed using price quotes from industrial suppliers. For hazardous waste disposal, the average U.S. prices varied from $3 to $4/kg waste disposed based on disposal methods of landfilling and incineration (*21*). For the rest of the chemicals, only point estimates were available, and therefore, ±20% variation was used to arrive at the upper and lower bounds. The cost of recycled PDK resins was based on cost of recycling different types of waste plastics and ranged from $0 to $1.23/kg based on 2019 prices reported by U.S. recycling (*25*). Material recovery facilities may be incentivized to add a new material stream and sort waste if the recovered material yields a higher price. Therefore, we use two currently high-priced waste materials (natural HDPE and premium PET) as proxies to account for a potential high purchase price for PDK and its consequent effects on MSP. Each parameter is provided in tables S1 to S5.

### Life cycle GHG emissions for primary and circular PDK resins

**Fig. 3:**
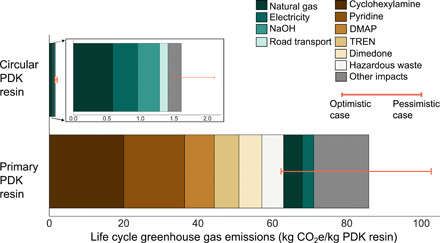
Life cycle greenhouse gas contributions from primary and circular PDK resins. For clarity, the inset adjoining the circular PDK bar represents magnification of the breakdown. The stacked bars are for a baseline scenario breakdown, and the error bars represent final values for pessimistic case and optimistic case. DMAP, 4-(dimethylamino)pyridine; TREN, *tris*(2-aminoethyl)amine.

Our framework tracks the life cycle of PDK resins starting from raw material extraction through production, ending at the facility gate. The end-of-life system boundary begins at the point when clean PDK waste leaves a sorting facility and is transported to a centralized production facility for chemical recycling to monomer. The system boundary excludes emissions associated with waste collection and sorting because these activities cannot be reliably allocated to PDK waste, and the nature of these activities will vary with the level of source separation or take-back programs in place. Figure 3 depicts the life cycle GHG emissions associated with circular PDK resins as compared with primary PDK resins. We estimated the life cycle GHG footprint of primary PDK resins to be 86 kg of CO_2_ equivalent per kilogram of product. The production of circular PDK resins results in emissions of only 1.6 kg of CO_2_ equivalent per kilogram. For primary PDK resin production, 48% of the GHG footprint is from the use of cyclohexylamine, pyridine, and chlorosulfonic acid, which are all chemicals required for the synthesis of DCC. Compounds such as DMAP and TREN that contributed to a higher price also contribute to life cycle GHG emissions, with DMAP contributing 9% and TREN contributing 7% to the total GHG emissions. The emissions associated with waste management (dominated by hazardous waste incineration) account for 6% of the total life cycle GHG emissions.

For circular PDK resins, the GHG emissions are substantially reduced as we eliminated use of aforementioned raw materials completely. Natural gas combustion comprises 36% of the life cycle GHG emissions for circular PDK resin. The estimate includes natural gas used in the entire life cycle, including both direct use at the facility for heat and power and indirect use in the upstream supply chain for raw materials. From material standpoint, NaOH required for TREN recovery in ion exchange process contributes 20% to the total GHG emissions. While NaOH is not an expensive material, it has a comparatively large life cycle environmental impact (~1.4 to 2.7 kg of CO_2_e per kilogram of NaOH). The road transport for circular PDK resins refers to the transport of waste PDK from collection/sorting facility to the recycling/production facility. We assumed a transport distance of 366 miles from material recovery to recycling based on industry-reported values for PET recycling (*26*). This distance represents a weighted average of reported distance from material recovery facilities (primary sorting), plastic recovery facilities (secondary sorting), and deposit centers. As it is unclear whether PDK waste supply chains would require longer/shorter distance transport depending on levels of sorting (e.g., a further secondary sorting may increase the total distance traveled), we also use a 20% variation in distance for sensitivity analysis. We should note that, usually at a longer distance, multimodal transport involving a combination of truck, train, or ocean transport may be more favorable and would likely reduce overall emissions (*27*). For the sake of simplicity, we limited the analysis to truck transport and used conservative estimates for emissions. The model includes an on-site electricity and heat generation plant using a natural gas–fired steam turbine. Therefore, the electricity use indicated here is separate from on-site generation and refers to indirect electricity used in the life cycle of circular PDK. The rest of the contributions are from chemicals used for circular PDK resin production as well as solid waste and wastewater treatment. To estimate total life cycle GHG emissions, we used publicly available LCA databases, process engineering estimates when industry data are not available, and a hybrid process-based/physical input-output (IO) approach to conduct the inventory modeling. We used sensitivity analysis to capture resulting variation in our estimates. In Fig. 3, the breakdown by major contributor is presented for the baseline case, while the sensitivity bars represent the pessimistic case and optimistic case. Similar to techno-economic analysis, at least three data points of low, average, and high values were collected when possible, and in the absence of a range, point estimates and their ±20% variation were used. A complete visualization of the life cycle impact contributions is presented in fig. S1.

**Transitioning from primary to circular PDK resins in sustainable manufacturing**

We modeled a mixed PDK resin plant comprising of both primary and circular PDK resins. The plant takes in increasing amounts of PDK waste each year it is in operation to produce circular PDK while coproducing primary PDK resin from raw materials as makeup. The total production at the individual facility modeled here is targeted at 20,000 MT. The goal of this scenario was to understand how quickly costs can be leveled by transitioning from primary to circular resin production in sustainable manufacturing as it depends on product lifetime and recovery rate. To this end, we constructed a series of logistics scenarios to capture the expected lifetime and recycling rate for different product classes for PDK waste recovery.

As PDKs can potentially replace several different polymers in the market, we selected two general use-phase scenarios to assess their effects: PDKs used in packaging products and PDKs used in consumer and institutional products. The distinction between types of different uses is important as it dictates the time lag between production and potential waste recovery. For example, packaging materials are recovered faster (average residence time in use phase of 6 months) than consumer products (~3 years). Product residence times in the use phase may vary depending on the location, demographics, and individual recycling practices (*28*). We used log-normal distribution to capture these associated uncertainties. The product categories, their residence times, and assumed distributions are derived from Geyer *et al*. (*8*).

Recycling consists of four major steps: transport, collection of waste, sorting in a recovery facility, and final recycling operations. In the absence of an established recycling supply chain for PDKs, we use two recovery rates to represent potential material loss during the process. The 100% recovery represents a “theoretical maximum” and, therefore, an extremely optimistic case where all PDK waste is recovered each year subject to the time lag (based on residence time) for specific products. The 44% recovery case represents a baseline case for year 2050 based on extrapolation of current recycling trends estimated by Geyer *et al*. (*8*). Here, the term “recovery rate” of 100 and 44% only refers to collection of waste in waste recycling supply chain. This is different from recycling yields for circular PDKs (e.g., 1 kg of circular PDK requires 1.26 kg of waste PDK). Therefore, 100% recovery rate will only supply 20,000 MT of waste PDK to the refinery, and makeup material will be required to meet the material losses inside the refinery.

**Fig. 4:**
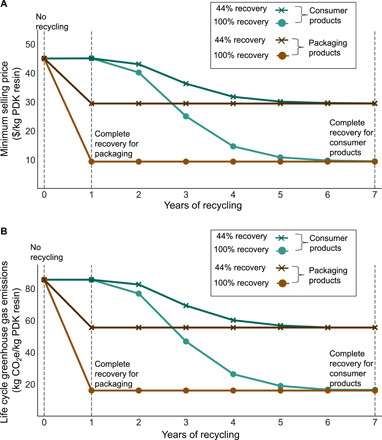
Leveled cost and GHG emissions curves by year of operation, including recovery and recycling of waste PDK as products and packaging reach their end of life. (**A**) Leveled cost curves and (**B**) GHG emission curves as a function of years of operation incorporating increasing amount of waste collected from previously sold PDK-based products. The curves depict different % product recovery. A 100% recovery refers a situation of no loss where all waste material collected is recovered. The collection is subject to lag time for product use and end of life modeled based on log-normal distribution. The vertical lines represent the year complete product recovery occurs for different use phases.

The years of recycling in Fig. 4 indicate each year PDK resin in the market is recycled back into the system for depolymerization. In year 0, we assume that there are no PDK products in the market, and thus, none is recycled back into the facility. From year 1, the collection and recovery of PDK waste progress as more waste is collected and recovered from the market. The collection rate is estimated by combining the previous year’s production of 20,000 MT with useful life of a product modeled based on a log-normal distribution. At year 7 for consumer products and year 2 for packaging, a majority of PDK waste is collected (19,944 MT for 100% recovery and 8776 MT for 44% recovery) from the market and reaches a steady state in subsequent years. As seen from Fig. 4, with 44% recycling, the circular PDK resins do not reach a price point below $30 or GHG footprint of 56 kg of CO_2_e per kilogram of resin. With 100% recycling, the mixed PDK resin production reaches an MSP of $10/kg of resin and GHG footprint of 17 kg of CO_2_e per kilogram of resin. We note that even with 100% recycling at a mixed facility, an MSP comparable to a standalone, purely circular PDK processing facility cannot be achieved. This discrepancy results from a number of factors: The material and efficiency loss in the refinery require additional makeup material (DCC, DMAP, dimedone, and TREN) to produce makeup primary PDK for a total production of 20,000 MT of circular PDK. Furthermore, there are additional capital and operations cost of setting up a mixed PDK facility including both primary and circular productions. As seen from Fig. 4, more realistic recovery rates will affect the results, increasing the cost and GHG emissions by requiring increased amount of makeup primary PDK. Therefore, apart from boosting recovery rates, improving the process chemistry will be necessary for leveling the cost and emissions. In terms of selecting product use, PDKs in packaging material can enable a complete recovery earlier in the recycling cycle and reduce the payback period, but use for packaging provides a lower profit margin, requiring more economical production. Modeling calculations associated with scenario analysis are provided in table S6.

### Comparison with commodity polymers

**Fig. 5:**
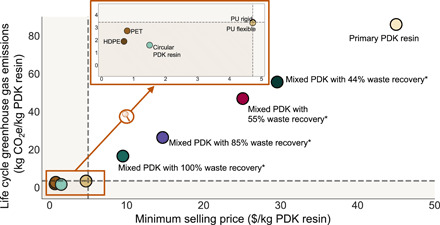
Comparison of life cycle GHG emissions and minimum selling price of PDK resin with GHG emissions and bulk selling prices of commodity polymers. Inset: Polyethylene terephthalate (PET), high-density polyethylene (HDPE), and polyurethane (PU). The GHG emissions for commodity polymers represent cradle-to-gate system boundary. *Mixed PDK resins consist of circular PDK resin produced from X% recovery of waste PDK resin and the makeup produced from virgin materials for a 20,000 MT manufacturing plant.

To assess whether PDKs can compete with their commercially available counterparts, we compare costs and GHG emissions of HDPE, PET, and polyurethane with PDK resins in Fig. 5. Mixed PDK resins with 100% recovery, 85% recovery, 55% recovery, and 44% recovery are shown where the given percentage of waste PDK is recovered (from 20,000 MT of PDK annually produced), and the remainder is sourced from raw materials to produce a total of 20,000 MT of PDK resin. For simplicity, we do not make any assumptions regarding product use or time lag of product diversion in the market. In Fig. 5, the shaded area represents maximum GHG emissions and cost from commodity polymers. The inset represents a magnified version focusing on commodity polymers for legibility. HDPE and PET serve as representative for packaging and polyurethane for consumer goods. The bulk prices for these materials were obtained from Alibaba (*29*) and the GHG emissions from the literature (*30*, *31*). A detailed list of sources for commodity polymers’ emissions is provided in the tables S7 to S9. We note that the life cycle GHG emissions of commodity polymers cannot be compared to one another as they are not functionally equivalent, but PDK resins are a viable replacement for each in some applications.

As shown in Fig. 5, circular PDK resins outperform HDPE, PET, and PU rigid/flexible on the basis of GHG emissions and outperform only polyurethane on price. Mixed PDK resins, which represent a more realistic near-term scenario, cannot match either the price point or GHG emissions of commodity polymers despite increasing waste recovery rates. Even with 100% recovery and collection amounting to 20,000 MT of PDK waste per year, a small amount of makeup raw material is required to compensate for efficiency losses in manufacturing, and this increases the cost and GHG emissions. For context, mechanically recycled PET emits 0.91 kg of CO_2_e per kilogram of resin, while recycled HDPE emits 0.56 kg of CO_2_e per kilogram of resin. Previous work has shown that mechanical recycling is generally less energy and emissions intensive than chemical recycling (*19*). However, it is difficult to directly compare mechanically recycled plastics with circular PDKs because functionality may differ as one can only be recycled a few times before disposed while PDKs can be theoretically recycled infinite times. These results demonstrate that, while circular PDK resin technology is promising, eliminating high-cost and GHG emission–intensive material inputs during primary PDK resin production will be critical to its success and environmental benefits. Specifically, exploring alternative formulations for the triketone monomer that can eliminate use of DCC and DMAP and follow principles of green chemistry can make a substantial and potentially market-differentiating change (*32*).

## DISCUSSION

There is a growing consensus that commodity and specialty polymers and the manner in which they are used require a fundamental redesign with their end-of-life management more fully considered. PDKs and related depolymerizable polymer networks are compelling in some respects; a material that is costly to produce but recyclable at a fraction of the original cost provides a strong economic incentive for waste recovery, and the possibility of infinite recycling can further close the loop. However, novel polymers must achieve sufficiently competitive prices and environmental impacts to enable market uptake, and their recovery for reuse requires extensive upgrades in sorting and recycling infrastructure. The framework we present, which incorporates LCA, techno-economic analysis and materials flow scenarios, can serve as an approach to more fully capture the impacts of new polymers in circular manufacturing systems over time and the challenges of scale-up. With our analysis, we identified hot spots and bottlenecks in resin production. Specifically, for PDK resins, the economics and GHG footprint can be improved by transitioning away from the use of DCC, dimedone, and DMAP and making use of catalytic processes that generate less waste. Specifically, more work is needed to explore triketone synthesis pathways using less hazardous chemicals and still retain high yields (*33*). In addition, the scope of the study limits our analysis to cost and GHG emissions of hazardous waste disposal. While high values in these metrics reflect the environmental impacts of hazardous waste disposal, future work should focus on including emissions to soil, groundwater, surface water, wildlife, and air emissions such as volatile organic compounds to motivate further adopting green chemistry principles.

While new materials can play an important role in enabling a more circular economy, each component in the product supply chain (producer, consumer, and waste processor) must participate and successfully coordinate with one another (*34*). We find that, given the high recovery rates needed to lower system-wide costs and emissions, successful production of circular PDK resins hinges on strengthening the connections between the consumer and waste processing nodes of the system. Specifically, a waste processing node such as a material recovery facility may require retrofitting and upgrades to handle a new material and may only do so if a case is made for high recovery price as well as high volume to sort. While we account for higher price sensitivity by using the most expensive waste products as a proxy, PDKs may still face a chicken-and-egg problem; high recycling rates require successful sorting and material recovery facilities will only add a new line if recoverable volumes are adequate to justify the investment.

In addition, a balance must be struck between using chemically recyclable resins in longer-lived consumer products with greater potential for take-back programs and short-lived packaging that may be more difficult to reliably recover and sort. The multiyear life span of many consumer products will delay a hypothetical industry’s ability to benefit from the low-cost, low-impact recycling process. We fully anticipate this to be feasible given the relatively short time frame these materials have even been disclosed. In the United States, currently, only lead acid batteries have sufficiently high recovery rates (99%) (*5*). The highest recycling rate of plastic packaging is for HDPE and PET bottles at 31 and 29%, respectively, which would not be sufficient to make circular PDK resins viable. We note that high recycling rate of lead acid batteries is partly due to the substantial core deposit fee and the ease of returning a used battery through a separate supply chain. While bottle deposit schemes have been shown to increase recycling rates, such a substantial deposit as with batteries may not be applicable for a material used in packaging and most other durable goods. However, a specialized supply chain involving product take-back initiatives along with consumer awareness and regulations may boost material recovery rates compared with current curbside recycling rates.

Another requisite for successful circular economy is the role of government regulations, policies, and incentives as drivers of change. Over the past few years, governments across the world have enacted a slew of legislation targeting plastics including building efficient waste collection systems, industry engagement, plastic bag bans, and requirements for recyclability (*35*, *36*). However, a more concerted effort to promote dialog between scientists, policy makers, product, and recycling industry regarding issues ranging from green chemistry for production, establishing requisite recycling infrastructure, appropriate labeling, sorting, and consumer awareness of disposal of multimaterial products is required for boosting recycling rates.

Last, our model is a result of assembling data from diverse sources and the use of engineering judgment and economic assumptions grounded in current markets. There exists an inherent uncertainty in modeling a product not already established at commercial scale. Therefore, our results should be taken not as final values, but the ranges provided should serve as an approximate evaluation of the product’s environmental and economic performance. In addition, we assume one-to-one displacement between primary and circular polymer resins because the recycling process yields material identical to the primary resin. However, actual product displacement is driven by market forces. The same is true for a circular resin’s displacement of a commodity or specialty polymer, which is uncertain given the limited property testing conducted to date. A rebound effect such as Jevon’s paradox may occur, initially driving the demand for more primary resin (*9*). Conversely, the high cost of primary resin production may incentivize companies to reduce their consumption through, for example, limiting the use of unnecessary packaging.

## MATERIALS AND METHODS

### Plant size

First, we established an appropriate plant size to scale experimental data for commercial production. We determined the impacts of economy of scale on the production cost per kilogram of PDK resin (figs. S2 and S3). Our analysis suggests that, beyond 5000 MT production, the cost of producing PDK resin only marginally decreases from $45/kg with increase in capacity. Therefore, on the basis of our analysis and considering similar commercial-scale chemical recycling plants (*37*), we selected the PDK resin production capacity to be 20,000 MT. To assess how circular PDK resins would perform when sufficient product supply and demand are established, we modeled a steady-state inflow of PDK waste by assuming mature future “*n*th plant” (*38*). The steady state represents a case where the total demand is sufficiently met by recovered PDK waste. This assumption aids in comparing the cost and GHG emissions of circular PDK resin with current commercial products.

### Primary PDK resin production

#### Triketone monomer synthesis

The triketone monomer required for PDK resin production is synthesized with dimedone and sebacic acid (*13*), where the reaction occurs in the presence of DCC as a condensation reagent and DMAP as a catalyst in dichloromethane. The triketone monomer forms along with *N*,*N′*-dicyclohexylurea as a by-product. The reaction occurs at room temperature for 4 hours. The crude triketone monomer is recovered by adding 3 weight % (wt %) HCl and subsequently recrystallized using ethyl acetate at 80°C. Here, recrystallization was carried out to estimate analytical purity and may be skipped in manufacturing without affecting final PDK purity. On the basis of the experimental results, we used 90% conversion efficiency for modeling purposes. *N*,*N′*-dicyclohexylurea is a known irritant and modeled as a hazardous waste with appropriate cost assigned for disposal. The monomer synthesis stage includes mixing tanks, a continuous blending tank, and a solvent recovery system involving filtration, centrifugation, and distillation for dichloromethane recovery. The schematic of process flow diagram with major process equipment is provided in figs. S4 and S5.

#### Polymer synthesis

The polymerization of triketone monomer occurs through spontaneous condensation, in this case monomer with TREN to form thermally processable PDK resins. We modeled TREN as an externally sourced chemical. In this case, the synthesized triketone monomer and TREN were added to a ball mill and milled for 45 to 60 min. The resulting powder was subsequently dried to remove water. The uniqueness of the chemistry lies in solvent-free synthesis that occurs at room temperature with no additional heat requirement. The dried powder is pressed at 20,000 psi pressure and 190°C temperature for 60 s to yield transparent PDK material of desired shape. The polymer synthesis stage includes mixing tanks for reactants, a ball mill, a drying unit, and a thermal pressing unit.

### Circular PDK resin production

#### Depolymerization

Recycling involves three steps: collection, sorting, and chemical/mechanical recycling. We modeled PDK resins as use-phase agnostic, and therefore, we did not make any assumption whether the collection and sorting would be postconsumer or postindustrial. We only consider the third step of chemical recycling and include transport of PDK waste from sorting facility to chemical recycling, and the chemical recycling/depolymerization facility in our system boundary. In the facility, PDK waste is hydrolyzed in 5.0 M H_2_SO_4_ to recover triketone and TREN monomers at room temperature for 12 hours. The crude triketone monomer was recovered via filtration and by solubilizing the solid with aqueous K_2_CO_3_ and was subsequently precipitated in the presence of aqueous H_2_SO_4_. TREN was recovered using a regenerative resin process where it underwent ion exchange in the presence of a strong anionic resin. We used 50 wt % NaOH for column regeneration. Because of an excess of H_2_SO_4_ used for TREN recovery and subsequent ion exchange process, our effluent contains ~340 g/liter Na_2_SO_4_ and accounts for 28 wt % of the final effluent. Such a high concentration would require either pretreatment (*39*, *40*) or valorization of the salt before the effluent undergoes traditional wastewater treatment. Particularly, treating Na_2_SO_4_ as a coproduct rather than waste may generate additional revenue opportunities including purifying and selling as is with the current market value of $100/MT (*41*), or further treated to potassium sulfate (*42*) and sold at a higher price of $414/MT as fertilizer (*43*). In addition, unreacted TREN and monomer are assigned to solid waste category to account for cost and emissions; however, our future work will explore additional ways to recover or minimize the efficiency loss. The depolymerization stage includes a storage tank for acid, a reactor for depolymerization, an ion exchange unit, and a vacuum filtration unit.

### Auxiliary facilities

For each of the plants producing primary, circular, and mixed PDK resins, we assumed that required energy and heat are generated on-site using a natural gas-fired boiler. On-site energy generation unit includes a boiler to generate steam, which is sent to an extraction turbine coupled with an electric generator. If additional electricity is generated, it is exported and sold to the grid. In addition, we modeled a separate utilities section that cycles cooling water with additional makeup cooling water and process water sourced from outside with an assigned cost.

### Mixed PDK resin production

To understand the impact of current recycling infrastructure on the quantity of PDK recovered, we modeled a combined plant that produced both circular and makeup primary PDK resin materials, which we termed as “mixed PDK resins.” We modeled the recycling of PDK waste each successive year and calculated the amount of makeup raw material required to produce 20,000 MT of resin consistently each year. To this end, we modeled several scenarios with each incorporating a combination of different recycling rate and product life span. We conducted this exercise to measure number of years and the level of recycling required to recover sufficient PDK waste for the plant so it primarily operates the depolymerization unit using PDK waste as the main input.

Before a product is available for end-of-life management, it may go through various stages of use, repair, refurbishment, and exchanging hands. The accumulation of material in the use phase is represented by product life span and can vary based on type of product, region of use, and demographic practices. Following Geyer *et al*. (*8*), we modeled product life spans as ranges represented by log-normal distribution. We explored two cases: one where PDK resin is used as packaging with a shorter average life span of half a year, and second, where PDK resin is used as a generic consumer and institutional product with an average life span of 3 years. Recycling rates represent the efficiencies in collection and sorting of waste and vary with products. For example, lead acid batteries have a recycling rate of 99%, and PET and HDPE plastics have a recycling rate of ~30% in the United States (*5*). For this scenario, we selected recycling rates of 100 and 44%. Our selection of 100% represents a theoretical maximum, and 44% represents a baseline case from 2050 projections for plastic recycling provided by Geyer *et al*. (*8*).

### Techno-economic analysis

The techno-economic model for a PDK resin production facility was developed using process simulation software SuperPro Designer (*44*). The facility operates 24 hours/day and 330 days/year. The plant produces 20,000 MT of PDK resin (primary, circular, or mixed depending on the specific scenario) annually. The plant life is assumed to be 30 years. The assumptions for calculating the MSP are based on techno-economic reports on similar precommercial processes for bioenergy conducted by National Renewable Energy Laboratory and provided in table S10 (*38*). The costs for production were estimated by including capital and operating costs. For capital cost contribution, the total cost of equipment was determined based on equipment purchase price, required equipment size, and number of equipment from the process simulation. We used an average multiplier of 1.7 with purchased equipment costs to determine the installed equipment cost. The capital cost estimates also include additional costs such as cost of setting up warehouse, site development, additional piping, project land contingencies, and permits. Each cost is approximated as a percentage of the installed and total direct costs, and the percentages are provided in the table S10. The annual operating costs include cost of material, maintenance, labor, transportation, and waste management and are obtained from SuperPro Designer. The prices associated with each raw material purchase and electricity were obtained from the literature (*38*, *45*) and e-commerce websites using request for bulk quotes (*29*, *46*). The cost of labor was estimated from the U.S. Bureau of Labor Statistics and the literature (*47*, *48*). The MSP was calculated by conducting a discounted cash flow analysis using a 10% internal rate of return. The assumptions regarding tax rate, internal rate of return, financing, and depreciation are further detailed in fig. S6.

### Life cycle assessment

The scope of the LCA is cradle–to–facility gate with a functional unit of 1 kg of resin produced, which is compared against 1 kg of a range of commodity and specialty polymers in use today. We also included the recycling process as it is an integral part of sourcing the raw material for circular PDK resins. The life cycle inventory data for input materials and commodity polymers are obtained from peer-reviewed literature (*49*, *50*) and LCA databases including ecoinvent (*31*), U.S. Life Cycle Inventory (USLCI) (*51*), The Greenhouse gases, Regulated Emissions, and Energy use in Technologies Model (GREET) (*52*), and Waste Reduction Model (WARM) (*53*). The life cycle GHG footprints of TREN and dimedone were estimated using neural network models that identified compounds with similar molecular characteristics and existing production data because no data specific to the production of these compounds were available (*54*–*56*). When these models could not identify any similar compounds, as was the case for DCC, we obtained their industrial synthesis route through available patents and created a separate process simulation to estimate material and energy use, which was then used to calculate life cycle GHG emissions (*57*–*59*). A detailed description of the modeling process for GHG emissions along with data sources is provided in the section S3. Next, we combined the data gathered in the life cycle inventory with the Intergovernmental Panel for Climate Change 100-year global warming potential characterization factors to arrive at final life cycle GHG emissions. We used a hybrid LCA approach that combined process-based model with IO matrix using physical units and created a process matrix to compute total requirements for production (*60*, *61*). The hybrid approach overcomes the disadvantage of the economic sector aggregation issues of IO modeling while still retaining the benefit of a larger system boundary and avoiding cutoffs in system boundary compared with traditional process-based LCA Because of the closed-loop nature of the process, we do not assign environmental burdens to PDK waste as recommended by ISO 14044 (*62*).

### Sensitivity analysis

To understand the variation in our results, we explored two cases: a pessimistic case and an optimistic case. The optimistic case represents a combination of lower prices, lower GHG emissions, and higher reaction yields. Similarly, the pessimistic case represents a combination of higher prices, higher GHG emissions, and lower yields compared with the baseline case. For the sensitivity analysis, we selected the dominant contributing factors for GHG emissions and MSP and varied the parameters based on available range of data or by varying the data by ±20%. The data are provided in table S4.

## SUPPLEMENTARY MATERIALS

Supplementary material for this article is available at http://advances.sciencemag.org/cgi/content/full/7/15/eabf0187/DC1
